# Imprecision in Vision: Lessons From Neural Circuits in the Fly

**DOI:** 10.1002/bies.70122

**Published:** 2026-03-03

**Authors:** Mathias F. Wernet, Marion Silies

**Affiliations:** ^1^ Fachbereich Biologie, Chemie & Pharmazie Institut Für Biologie – Neurobiologie Berlin Germany; ^2^ Institute of Developmental Biology and Neurobiology Johannes‐Gutenberg University Mainz Mainz Germany

**Keywords:** cell type, connectome, Drosophila, heterogeneity, synaptic connectivity, visual system

## Abstract

Visual systems appear like homogenous structures, where identical functional units repeat themselves across the eye. This architecture is thought to ensure a uniform sampling of the surrounding environment. Furthermore, anatomically and functionally identical properties of single units belonging to the same cell type, yet located across retinotopical positions are thought to ensure translational invariance. At the same time, regional differences and stochastic variations in microcircuit architecture have been linked to the processing of specific visual features. Recent access to connectomic datasets has revealed heterogeneity in visual circuitry that is at odds with these criteria: Cells considered to belong to the same type are variable in number and identity of connected partners, as well as in the relative number of synapses. This variable connectivity suggests that heterogeneous computations, even within defined cell types, is the rule, rather than the exception. It is therefore an exciting question whether these network properties increase functional variability, or even functional robustness, of visual processing.

## Graphical abstract

Visual systems are considered regular, retinotopic structures. Here, we summarize findings, including evidence from recent connectomes, that demonstrate cellular and synaptic heterogeneity in the Drosophila visual system —from regional specialization and stochastic photoreceptor patterns to heterogeneous synaptic connectivity. We discuss how this heterogeneity may produce functional diversity and robustness of visual processing.

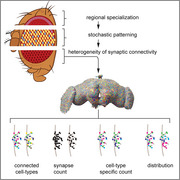

## 1 Introduction

When we navigate our environment, incoming visual information remains stable and thus robust to different viewing conditions. These include differences in brightness, differences imposed by self‐motion, changes in viewing angle, and distance. Hence, we perceive the shape, color, or contrast of an object, such as a tree, the same, even when the projection of the tree on our retina changes, for example, while we move past this tree, or when a cloud casts a shade on it. Our visual system thus produces the same output despite a shift in input position, which we refer to as translation invariance. This translation invariance must be a common feature of both vertebrate and invertebrate visual systems. While translation invariance can be explained by the homogeneous organization of visual systems, we will here discuss recent evidence for a remarkable degree of cellular and synaptic heterogeneity that argues against uniform function across the visual system.

As a basic principle, visual systems are composed of repeating units, each of which processes the same information about the visual world, yet sampling different points in space [1]. This mosaic‐like, periodic tiling of the visual input forms the basis of uniform information processing across the eye and makes vision robust to translational shifts. The classical view is that, within each of the repeating units, which we will refer to as columns, the same set of neuronal components processes light captured by photoreceptors to extract increasingly complex visual features along the visual hierarchy. These repeating columns and their neuronal circuits are composed of a set of distinct cell types that are typically described by anatomical, physiological, and genetic criteria. Anatomical criteria encompass the cellular morphology and dendritic or axonal projection patterns of a neuron, which all contribute to making cells of one type **anatomically uniform** and distinct from other cell types. Furthermore, many cells in the visual system tile the physically available space, meaning that the dendrites of all cells of one type together cover visual space, yet the dendrites of adjacent cells of the same type do not overlap. Whereas all of this anatomical evidence argues for uniformity, **some anatomical features are heterogeneous**, and deviate from this uniformity. This ranges from the stochastic organization of photoreceptor subtypes in the retina to the variable number or projections of cells of one type [2, 3]. With such heterogeneous anatomical properties, functional translational invariance can be achieved despite, or even because of deviations from highly uniform processing across the eye.

Cells of one type are considered **functionally uniform** based on their physiological properties: This begins with cells responding either selectively to only ON or OFF contrast and extends to more specific, temporal characteristics of a cell type [4, 5, 6, 7]. Both ON and OFF visual pathways, for example, contain neurons with a distinct set of temporal filtering properties, some faster, some slower. Through combinations of neuronal inputs with distinct spatial and temporal properties, downstream neurons can become selective for certain motion directions [4, 5, 6]. Functional features like contrast selectivity, spatial and temporal receptive fields, spatiochromatic receptive fields, or direction selectivity are all physiological hallmarks that can help define a cell type [7, 8, 9]. Nevertheless, **heterogeneity exists within the physiological properties** of each cell type [10, 11, 12, 13], more in some than in others, arguing that physiological heterogeneity is functionally meaningful.

Finally cells of one cell type are also thought to be **genetically uniform**. They express a common set of genes and can therefore be labeled by marker genes (or their promoters), which distinguish them from the expression profile of other cell types [14, 15, 16]. This helps to both classify and genetically target cell types. Over the past decade, single‐cell RNA sequencing data have contributed to this genetic classification of visual cell types [12, 17, 18, 19, 20] and the visual system in particular has helped to benchmark this technology to group cells into transcriptomic clusters that belong to one type [21, 22]. However, transcriptomic profiles can be more or less discrete, arguing for **transcriptional cell type heterogeneity**.

Thus, heterogeneity exists regardless of the criterion used for grouping neurons into cell types. Furthermore, examples exist where anatomical, functional, and genetic criteria do not align, such as in a recent study that combined transcriptomic profiles, function, and morphology of cells in the zebrafish optic tectum [23]. These observations fall in line with a recent discussion of neural cell types being fuzzier than classically viewed, such that even cells that have been described to belong to one cell type can diversify in their molecular, physiological, or as we will focus on in this review morphological properties [24].

Everything discussed so far applies to all visual systems. Anatomically different cell types have arguably been the most comprehensively characterized in the visual system of the vinegar fly, *Drosophila melanogaster*. It links a well‐described visual system architecture to a range of sophisticated visual behaviors, which are as diverse as walking, flying, learning, escaping threats, or pursuing another fly when attempting to mate [25, 26, 27, 28, 29]. Importantly, specific genetic tools have been developed for targeting, many of the visual cell types. For example, large collections of genetic driver lines exist [30, 31, 32] which can induce the expression of any effector or reporter gene in—in principle any cell type of interest. These allow visualizing cellular morphology, measuring neuronal activity, and ectopic activation or inhibition of a neuron [33, 34, 35, 36, 37].

Cell‐type‐specific genetic driver lines were also used to specifically label, isolate, and thereby assess the transcriptional profile of individual cell types. These, together with single‐cell datasets, allow for bridging genetic, neuroanatomical, and functional analysis for cell‐type‐specific analysis relatively easily. Given the considerable functional heterogeneity within different cells of one type, even within the periphery of fly visual system [10, 13, 38], the question arises how this heterogeneity aligns with the concept of uniformity across a visual system's retinotopic structure? With recent advances in connectomics one can now quantify anatomical similarities or differences between cells of one type, at a level of precision that was impossible before. A connectome grants access to all cell types of the visual system and provides information about their synaptic connectivity across all retinotopic units of the entire eye. Below, we will integrate recent connectomic data with previous analyses of cell types across the fly visual system and review what we have learned about regional specializations vs. unit‐by‐unit heterogeneity.

## 2 Homogeneity of Cell Types in the Fly Visual System

**FIGURE 1:**
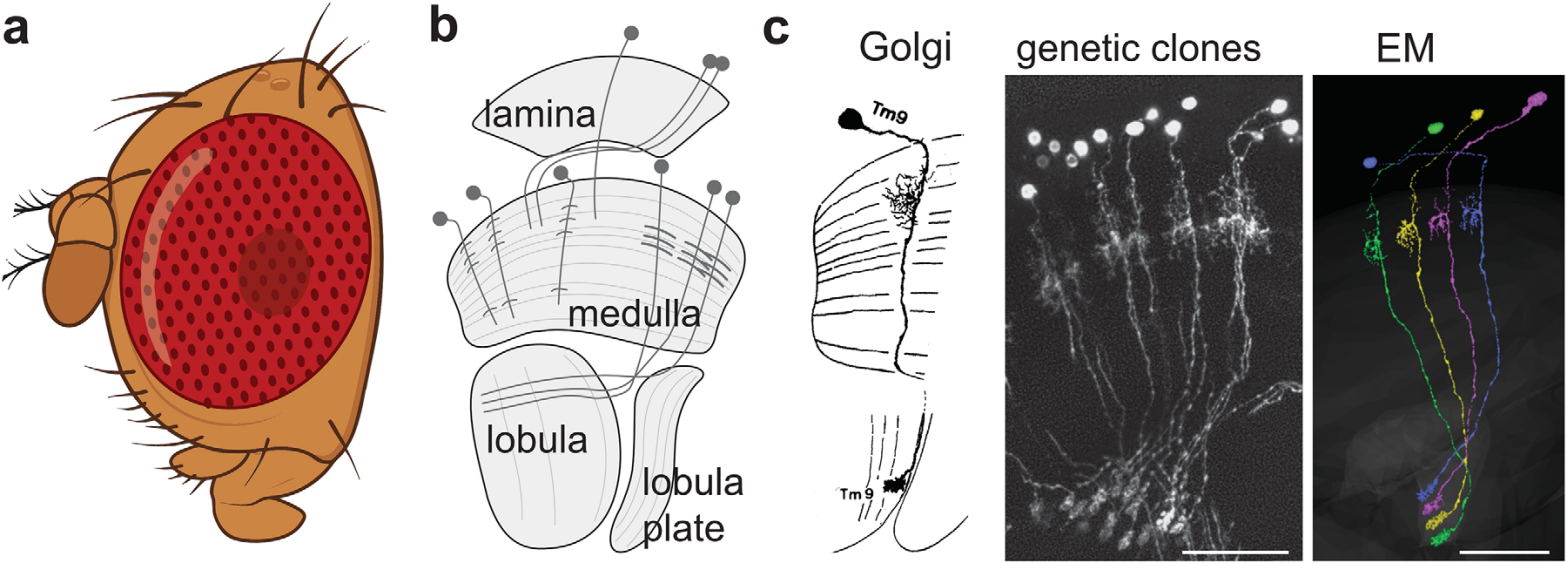
**Cell types in the fly visual system** (a) Schematic of a fly eye, Created in BioRender. Silies, M. (2026) https://BioRender.com/dgny2wp, modified with ChatGPT. (b) Schematic of the major neuropils of the Drosophila visual system, the lamina, medulla, lobula, and lobula plate, depicting some example neurons. (c) Cells of one cell type have uniform morphologies, allowing to assign neurons to a cell type across specimens and visualization methods. Shown are examples of Tm9 neurons, visualized via Golgi stainings (left, modified after Fischbach et al. 2019), genetically generated single cell clones using the Flp‐out technique (middle), or EM‐based connectomes (right, image produced in Flywire).

At first glance, fly eyes are homogeneous, regularly arranged, crystalline‐like structures (Figure 1A). An analysis of the morphology and architecture of circuits downstream of photoreceptors provided ample evidence for a highly homogeneous and repetitive organization. A detailed and very important first description of cell types in the fly visual system, based on the Golgi technique resulted in the classification of cell types based on their stereotypic and repetitive appearance, both within and across individuals [39]. Thus, cells could be grouped into one type solely based on their distinct dendritic and axonal stratification patterns (Figure 1B). 25 years later, these morphologies were confirmed via genetically marked single cells, for example, generated using genetic sparsening techniques (Figure 1C) [40, 41, 42, 43]. These light‐microscopic techniques added additional cell types (previously unstained by the Golgi technique), providing another important step towards a comprehensive description of all cell types in the fly visual system. More recently, this growing catalogue of cellular morphologies helped annotate cell types within electron microscopy‐based connectomes [32, 44]. Just the last two years have seen the release of the first full female and male visual system connectomes [32, 45, 46] vastly expanding previous connectome datasets that covered few columns [47, 48, 49, 50]. The annotation of cell types based on morphology and, recently, connectivity provided further support for the classification of many known cell types, and refined some cells into distinct of subtypes [32, 44, 46, 51]. As a result, anatomically defined cell types within the fly optic lobes, as well as their sex‐specific differences [52], are now known.

### 2.1 Heterogeneity in the Cellular and Synaptic Repertoire Across the Visual System

#### 2.1.1 Cellular Heterogeneity in the Peripheral Visual System

The above anatomical descriptors of cell types are predominantly defining cell types based on cellular shape. But what does connectivity tell us? Fly photoreceptors R1‐R8 send their axons to either one of two neuropils: the lamina (R1‐R6) or the medulla (R7 and R8). Within the first synaptic relay in the lamina, R1‐R6 connectivity to their retinotopic target structures is uniform and extremely precise. This is remarkable since photoreceptors from one unit eye (ommatidium) send their axons to six different neighboring units in the lamina, leading to a complex wiring pattern known as neural superposition. Almost no mistakes are made: there is only one mistargeting event in around 2500 photoreceptors analyzed [53, 54], and the initial patterning of photoreceptors can occur entirely independently of the target [55]. Each R1‐R6 photoreceptor forms characteristic synapses with four postsynaptic partners. This quartet always includes the same pair of lamina (L) neuron types, i.e. one L1 and one L2 neuron. Interestingly, the two additional postsynaptic partners can be either a third lamina neuron, L3, an amacrine cell, or a glial cell [56]. This illustrates a first example of heterogeneity in neural patterning of the eye at the synaptic level. The functional consequences of this non‐deterministic wiring pattern remain unclear. However, it is interesting to note that luminance‐sensitive L3 has recently been implicated in luminance gain control, which is in turn achieved by pooling luminance information across a patch in the visual scene [57, 58, 59, 60, 61]. Since luminance information of neighboring pixels is highly correlated in natural scenes [62, 63], and the average background luminance is used to normalize contrast computation [57], L3 does not necessarily need to contribute to this task in each visual unit. Contrast‐sensitive L1 and L2 neurons instead are the primary inputs to downstream neurons that compute local motion cues, which relies on spatially precise signals [4, 6]. Thus, precise, columnar connectivity might not be needed for some cell types (L3), as opposed to others (L1 and L2). The “freed up” connectivity can then be used by photoreceptors to also connect to other cell types (here: the amacrine cell, or a glial cell) and potentially serve additional functions.

**FIGURE 2:**
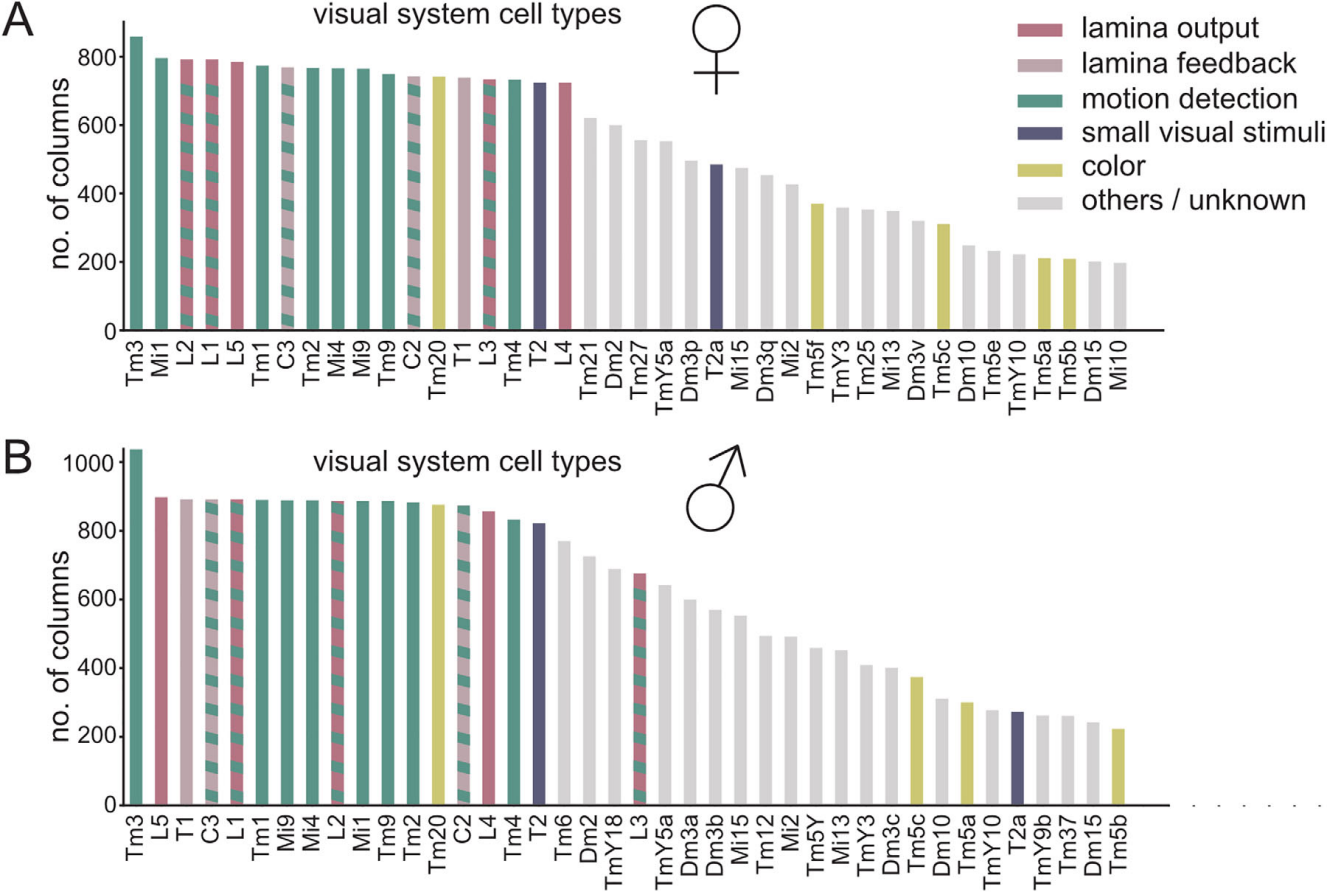
**Homogeneous and heterogeneous columnar presence. (a‐b)** Out of the top 40 visual system cell types shown here, which are postsynaptic to lamina neurons L1‐L5, ∼15 cell types are present once per column (~800 times) in both the female (data from [46, 70]) **(a)** and male (data from [32]) **(b)** fly brain. This list encompasses cells with known function (color coded). Please note the prevalence of cells with a known role in motion detection within the first 16 cell types.

Processing of visual information diverges into many parallel pathways downstream of the lamina neurons L1‐L5 (L4 and L5 receiving no direct photoreceptor input), with these five cell types synapsing onto more than 50 downstream cell types within the medulla [32, 45, 46]. Of these, only 15 cell types are described as being truly columnar [32, 64], virtually always being present once per medulla column, thus maintaining the retinotopic organization (Figure 2). The 15 columnar cell types encompass most of the lamina output (L1‐L5) and neurons and almost all major neurons of motion‐detection pathways in the medulla, namely the four major OFF pathway neurons Tm1, Tm2, Tm4, Tm9, and the major ON pathway medulla neurons Mi1, Mi4, Mi9. The fourth neuron type in this list of core ON motion detection circuits, Tm3, even slightly surpasses the total number of retinotopic columns (Figure 2), potentially because it is located at the edge of the columnar volume. The remaining columnar types are lamina feedback neurons (C2 and C3) (Figure 2), which have recently been implicated in tuning motion computation through inhibition [65], and another potential feedback neuron (T1) whose function is not well understood [66, 67]. Tm20 is the only columnar neuron assigned to pathways processing spectral information [68]. Finally, columnar T2 and T3 are small‐object‐sensitive neurons [69]. Thus, the function of most, if not all, truly columnar neurons is well understood and largely related to the computation of visual motion, which requires comparing signals from neighboring points in visual space. Vice versa, the function of most noncolumnar remains unknown. Here, it is important to note that noncolumnar neurons can in principle still tile the entire visual system, by having dendritic branches that extend beyond a single column. Their dendritic fields can even overlap heavily, especially if they surpass a certain size [32, 43, 44]. In this case, they will still pass on visual information to their downstream partners in fewer than all units of the fly eye, and thus with reduced spatial resolution.

Overall, it appears that most computations performed by cell types that are located in the periphery of the optic lobes, implemented by lamina neurons and neurons involved in local motion computation, have evolved to be largely uniform across the visual system. In contrast, considerable heterogeneity exists for the presence of all other (noncolumnar) cell types. This imprecision in spatial coverage could be tailored to specific computations requiring less precise spatial information.

#### 2.1.2 Stochastically Defined Retinal Cell Fates and Their Synaptic Circuitry

Considering that surprising variability exists even within peripheral circuits, what do we know about synaptic connections for which variability can be expected based on the stochastic specification of their circuit elements? Despite its homogeneous appearance, the adult fly retina consists of a retinal mosaic where at least two ommatidial types (historically named pale and yellow) are randomly distributed in an evolutionarily conserved, uneven ratio [71]. Due to different Rhodopsins expressed in photoreceptors R7 and R8 in pale vs yellow units, this retinal design appears well suited for color vision [72]. Since the discovery of the molecular mechanisms behind shaping this stochastic retinal mosaic, one could not help but wonder if the same downstream circuit element connects to both of these stochastic inputs or whether different cell types exclusively connect to pale or yellow inputs. Early light microscopic studies indeed revealed the first examples of medulla cell types being pale‐, or yellow‐specific [68]. Subsequent connectome analysis then unearthed several examples of cell types that showed a preference for forming synapses with either of the two stochastically distributed photoreceptor inputs [32, 68].

This raises the question how postsynaptic partners reliably connect to mixed, stochastically determined inputs. This question was partly answered when heterophilic cell surface molecules of the DIP/Dpr families were identified in R7 cells, as well as their targets: yR7 specifically express Dpr11, while their post‐synaptic Dm8 specifically express the heterophilic partner DIP‐gamma targets. However, DIP‐gamma negative Dm8 cells also exist, and connect to pR7 [73, 74]. Dpr11/DIP‐gamma binding during development is necessary only for the predetermined yellow Dm8 cells to suppress apoptosis; hence, wrong pairings and supernumerary target cells are eliminated due to the lack of a survival signal [75]. Surviving (pale) Dm8 cells in mutants lacking the DIP/Dpr signal readily form synapses with both pale and yellow R7 photoreceptors. This points towards a cellular sorting mechanism during development, by which pre‐ and post‐synaptic partners form synapses based on adjacency and competency, rather than strictly by a molecular code [76]. The question how variable these connections are when compared to non‐stochastic columnar cell types still needs to be addressed.

Although Dm8 is a multi‐columnar cell type, pooling inputs from up to 14 neighboring R7 axon terminals, the most intimate contact with the highest synapse count is formed in an anatomically distinct column, which is located towards the center of Dm8's dendritic tree. In this so‐called ‘home column’ the pale/yellow specific pairing occurs via Dpr11/DIP‐gamma [77]. In contrast, mixed pale/yellow R7 inputs in the periphery of any given Dm8 arbors form a variable ‘surround’ to the pale or yellow center [78, 79, 80]. It turns out that stochastic specification of R7 inputs might involve separate mechanisms for generating both stereotypic (pale‐pale or yellow‐yellow matching) and variable outcomes (variable pale and yellow surround). Since the total number of R7‐to‐Dm8 synapses varies significantly across ‘home columns’, it will be exciting to quantify whether these DIP/Dpr‐matched connections are more or less variable than the columnar ones described above, previously believed to be uniform.

#### 2.1.3 Regional Variations in Cellular Diversity and Synaptic Connectivity

A retina generating 800 retinotopic pixels of the visual world with columnar circuits along the visual hierarchy bears the advantage that the heterogeneous cell types of certain columns can be recruited to perform distinct computations. In addition to the stochastic distribution of photoreceptor types discussed above, such computations can for instance be achieved in a regionally restricted manner. This facilitates the appropriate encoding of stimuli that only exist in a specific part of the visual panorama, such as cues from the sky or a mating partner in front of the fly. Regional differences have been defined by different approaches. For example, scRNAseq analysis has suggested the emergence of regional subtypes, such as distinct dorsal and ventral populations of Tm9 cells, or regionally restricted T4 cells that are defined by marker gene expression [18]. These hint towards specializations across the eye that we do not fully understand yet. Anatomical analysis of the the fly visual system has provided two famous examples of such deviations from the standard columnar Bauplan, resulting in circuits where form dictates function.

**FIGURE 3:**
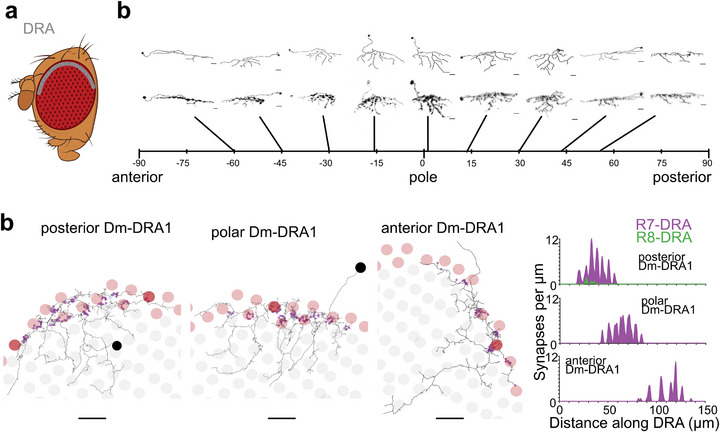
**Regional variations in morphological diversity and synaptic distribution. (a)** Schematic of a fly eye depicting the location of DRA ommatidia (grey). (b) Morphological variability of DRA‐specific Dm‐DRA1 cells in the medulla challenges traditional definitions of cell types (adapted from Sancer et al., 2019). Scale bars are 7 µm. Individual cells have been rotated to be horizontally aligned for better comparison. (**c)** Connectomic analysis reveals considerable variability in both morphology and connectivity within one cell type (adapted from [78]). All cells are oriented with dorsal pointing towards the top, with the anterior Dm‐DRA1 cell being closest to the equator.

In flies, a narrow band of ommatidia along the dorsal head cuticle is morphologically and molecularly specialized to detect the angle of linearly polarized skylight [81, 82, 83]. Like in many other insect species, the transition between these ommatidia in the ‘dorsal rim area’ (DRA) and the rest of the retina is formed by a sharp boundary, meaning ommatidial fate changes abruptly from one retinotopic unit to the next [84, 85]. The circuits downstream of the DRA compute skylight polarization for navigation, as was shown using behavioral experiments, optophysiology, light microscopy, and connectomic reconstruction [86, 87, 88]. DRA circuits are, in fact, different both in cellular composition and in synaptic connectivity—from their non‐DRA counterparts serving color vision. For instance, only in DRA columns, Dm8 is replaced by two distinct, yet developmentally related cell types, named Dm‐DRA1 and Dm‐DRA2, which connect to R7‐DRA and R8‐DRA, respectively [89]. This duplication of a Dm8‐like unit reflects the fact that only in the DRA, photoreceptor R8 adopts an R7‐like fate [85, 90]. The first characterization of Dm‐DRA1 cells challenged the classic anatomical definition of fly optic lobe cell types as it was described above. These cells manifest great morphological diversity within their own type, and gradually change their appearance along the dorsal periphery of the optic lobe (Figure 3A) [89]. While the functional significance of this unusual diversity remains unclear, preliminary connectomic analysis revealed that the specificity of R7‐DRA vs R8‐DRA photoreceptor inputs to Dm‐DRA1 cells were unaffected [78] (Figure 3B). The available connectomic data spanning three optic lobes (two female, [45, 46], one male [32]) will now allow for quantifying the consequences of morphological variability on synaptic connectivity.

Another well‐understood, locally restricted deviation from a reference Bauplan is the so‐called ‘love spot’ in the retina of the male house fly *Musca domestica*, and closely related species [91]. In this fronto‐dorsal region, the love spot is thought to be specialized for fixating on a female conspecific for chasing her during flight [92]. Only in this region, the photoreceptor R7 is transformed into an R1‐R6 counterpart, including a rewiring of the R7 axons to the lamina neuropil, the normal target area of R1‐R6. There, this ‘love spot R7’ now contributes to improved motion vision, at the expense of color sensitivity [93]. No such ‘love spot’ equivalent has been reported for the retina of *Drosophila*, which could reflect the fact that the vinegar flies pursue females while walking. No whole brain connectome exists for the species with a ‘love spot’, yet large male‐specific neurons have been reported downstream of it [94]. The *Drosophila* connectomes revealed a surprising number of new cell types that exist only in clearly defined regional zones, for which no particular function has yet been described [32]. Amongst them are male‐specific, frontally biased visual projection neurons, whose connectivity suggest that they constitute a functional love spot [52], indicating that circuit architecture is adapted to a specific function. This suggests that the locally restricted presence of specific cell types, such as visual projection neurons, could be a common theme for shaping neural computation.

#### 2.1.4 Different Synaptic Connectivity Patterns Introduce Heterogeneity in the Organization of the Visual System

**FIGURE 4:**
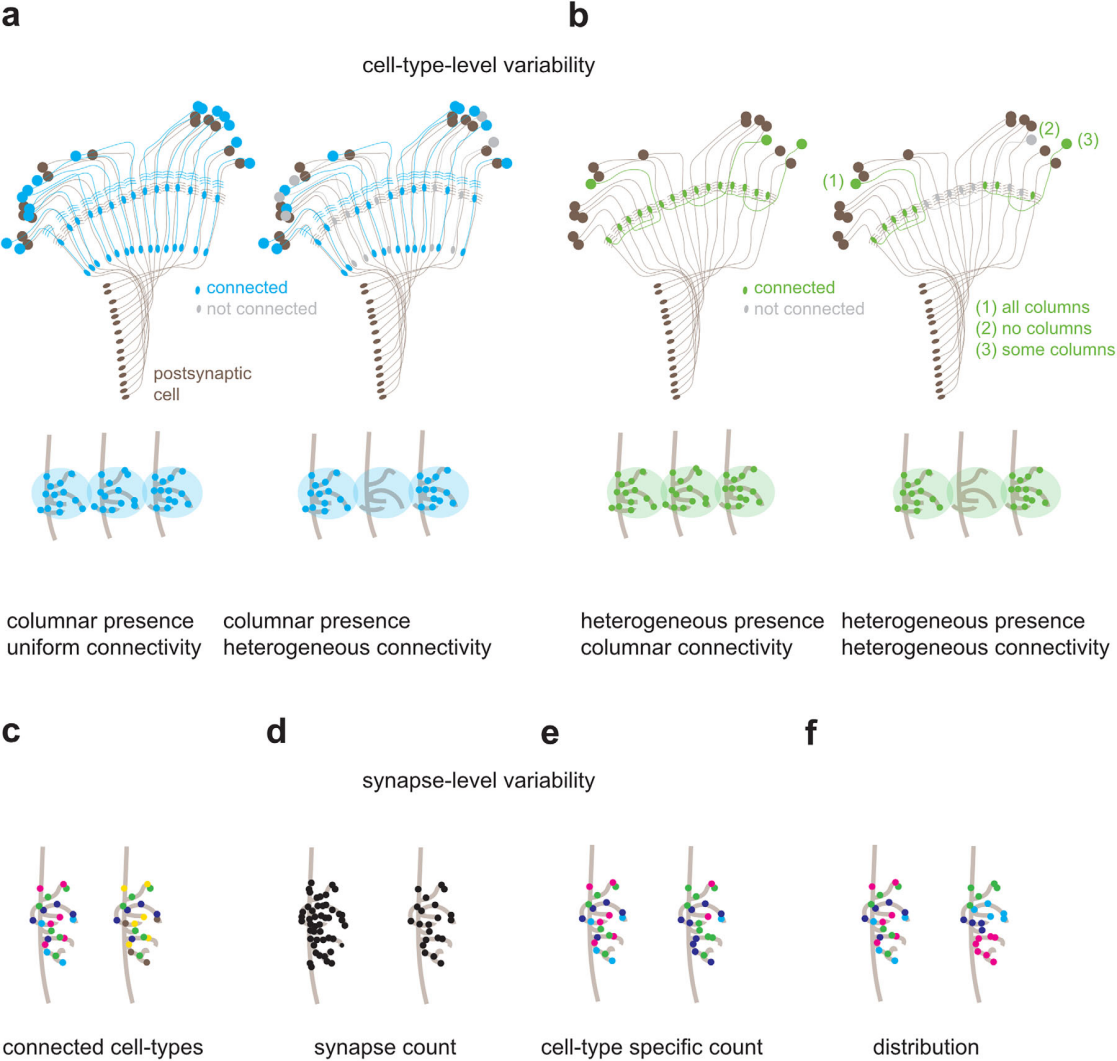
**Different types of heterogeneous wiring. (a, b)** Cell‐type‐level heterogeneity. (**a)** Columnar neurons (blue) can show uniform connectivity (left) or heterogeneous connectivity (right) to a postsynaptic cell (brown). Bottom: Illustration of three postsynaptic dendrites (light brown), which encounter a presynaptic axon terminal (light blue) and can or cannot form synapses (blue dots). (**b)** Same as in **(a)** for noncolumnar neurons (green), i.e., a neuron which is present less often than once per column, hence heterogeneously, but which can still extend branches in each column. Such cell types can be connected to a postsynaptic partner (1) in all columns, (2) in no columns, or (3) in some of the columns they branch across. **(c‐f)** Synapse‐level heterogeneity. (**c)** a postsynaptic dendrite (light brown) can receive presynaptic input from different cell types. (**d)** The overall or (**e)** cell‐type specific synaptic count can vary as well as the (**f)** spatial distribution of synapses across the dendrite.

From the examples above, it becomes clear that variability in the visual system is achieved at very different levels, from regional specialization, via heterogeneity in columnar presence, to heterogeneity in synaptic connectivity. Connectomic data now allows to look at the latter in more detail (Figure 4). As discussed above for the photoreceptor‐to‐lamina neuron example, columnar cells can show either uniform or heterogeneous connectivity to other columnar cells (Figure 4A). Conceptually, one can argue that non‐columnar cell types could also manifest heterogeneous presynaptic connectivity, meaning that the population of cells belonging to the same type does not form synapses in each retinotopic column. From the perspective of a single presynaptic cell, this could happen either through only some branches of a multi‐columnar neurons synapsing onto a specific postsynaptic cell type (Figure 4B, cell 1), or via entire neurons of the same type randomly choosing to form synapses onto a specific downstream cell type, or not (Figure 4B, cell 2/3). From the perspective of the postsynaptic neurons this would then lead to different combinations of presynaptic inputs that are variable both in the identity and number of inputs, as well as in their spatial distribution (Figure 4C). Whether and to which extent this is implemented remains to be studied.

Connectivity can also be heterogeneous at the synapse level (Figures 4C‐F): In addition to forming synapses with a different set of presynaptic partners (Figure 4C), heterogeneity of connectivity can in principle also extend to the amount of synapses that different pairs of the same pre‐and postsynaptic cell types form with each other (Figure 4D), or concerning the cell‐type specific amount of synapse that a cell makes with each of its synaptic partners (Figure 4E). Finally, the distribution of synaptic inputs along a given dendrite or other compartments can also differ, potentially shaping the information transfer onto the postsynaptic cell (Figure 4F). Since the functional role of most noncolumnar cell types it not well understood in the first place, the functional consequences of the heterogeneous aspect of their connectivity features are even less clear but can, in principle, impact neuronal function, either by making it more flexible, or more robust, which we will discuss further below.

**FIGURE 5:**
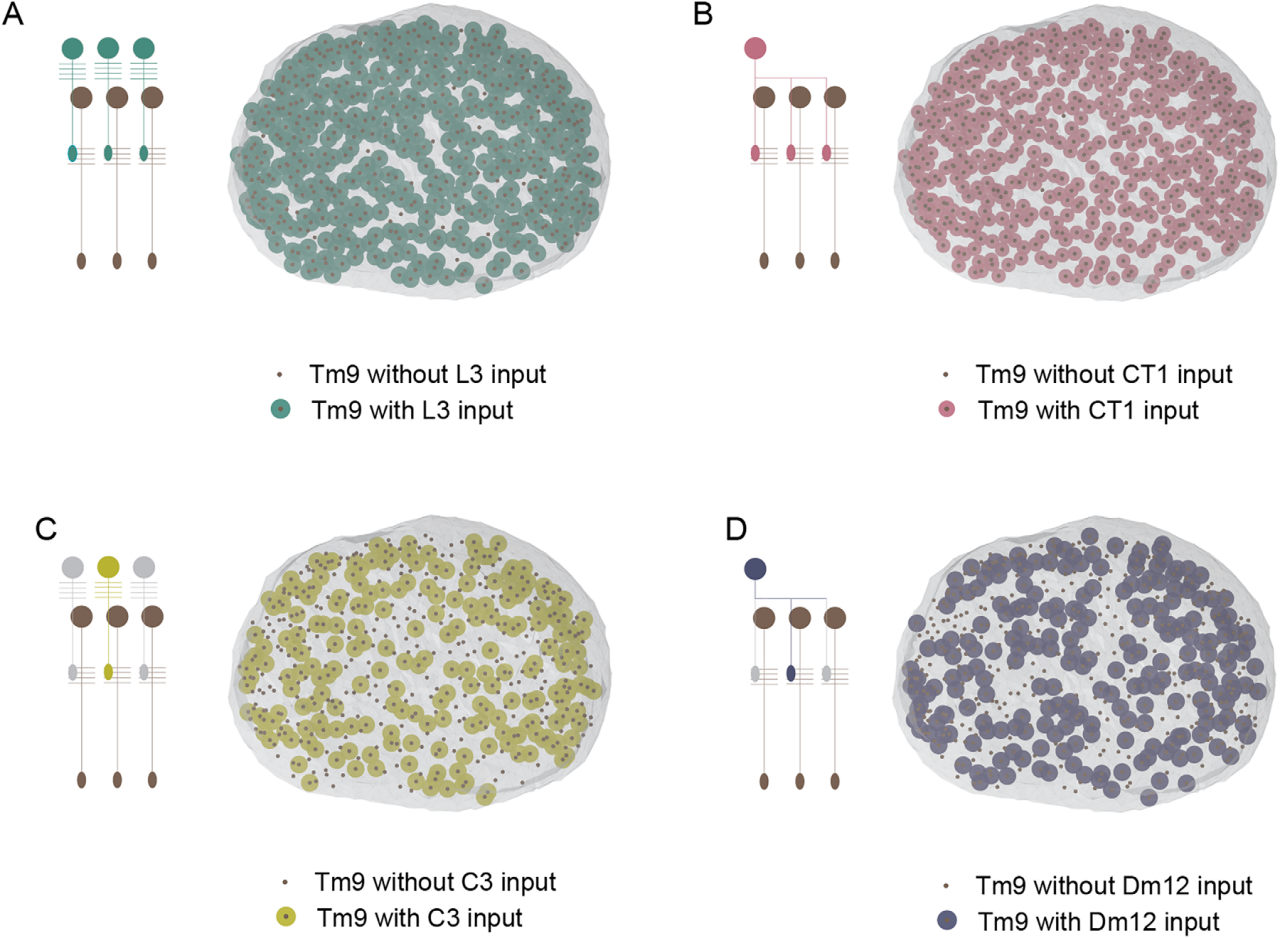
**Spatial distribution of connectivity patterns**. (**a‐d)** A postsynaptic Tm9 neuron is shown as a brown dot on a top view of the medulla neuropil. Presynaptic neurons are shown as large, colored circles only if they are connected to this postsynaptic neuron. Tm9 manifests uniform connectivity to (**a)** presynaptic columnar neurons (here: L3), or to (**b)** presynaptic non‐columnar neurons (here: CT1). The same neuron can show heterogeneous connectivity to **c)** presynaptic columnar neurons (here: C3), or to (**d)** presynaptic non‐columnar neurons (here: Dm12). (**c, d)** There is no apparent regularity in the spatial distribution of the heterogeneous presynaptic inputs. Note that this is the same type of data as shown in Cornean et al., 2024, but analyzed for (close to) all Tm9 neurons of a right female optic lobe, using Flywire. The schematics illustrate whether a presynaptic neuron is columnar/non‐columnar and if it shows uniform/heterogeneous connectivity, but do not show the true anatomy of the example cell types (C3 is a feedback neuron, CT1 is presynaptic to Tm9 close to its output region).

While an early study suggested that there is little variation in wiring between the columnar cells of 7 adjacent columns [64], this picture evolved with the emergence of many more annotated cell types and larger connectomes. A first detailed analysis of full visual system connectomes revealed that many of the above conceptual possibilities for achieving heterogeneous connectivity can be implemented combinatorially, by the same cell type. The columnar Tm9 cells of the OFF pathway for motion detection for example classifies as a cell type by many criteria: they are uniquely targetable by genetic driver lines, are present once per column, and show stereotypic morphology that distinguishes them from other cell types. Yet, Tm9 neurons manifest surprisingly few uniforms and many heterogeneous presynaptic partners when comparing synaptic connections across columns, which includes both columnar and non‐columar presynaptic cell types (Figure 4A and B). Tm9 uniformly connects to the columnar L3 and the non‐columnar CT1 cell (Figure 5A and B), and exhibits heterogeneous connectivity with other columnar and non‐columnar cell types, such as C3 and Dm12, respectively (Figure 5C and D). Furthermore, for both the uniform and the heterogeneous partners, the (absolute and relative) number of synapses varies for any given connected pair [10]. The spatial distribution of the heterogeneous connections appears to be stochastic, with no apparent pattern across the medulla and no apparent bias of synapse distribution along the dorso‐ventral or anterior‐posterior axis (Figure 5C and D). Thus, there exists no evidence for regional specialization or the emergence of specific circuit motifs. Instead, the connectome suggests that heterogeneous connections are stochastically distributed across the fly visual system.

Given that functional variability exists between Tm9 cells within one fly and across flies [10, 13], the heterogeneous connectivity patterns could account for this functional variability. This heterogeneity in connectivity and consequential functional heterogeneity would argue that not every column in the fly eye processes the same type of visual information. In this scenario, one could increase the functional repertoire of the visual system by ascribing several functions to the same neuron type. Such a circuit design might have evolved to handle a wide range of visual inputs with a limited number of cell types. Whether or not this is further constrained by the size of the visual system, remains to be seen.

Other, closely related cell types, e.g., Tm1 and Tm2, appear more stereotypic in their visual response properties. Connectome analysis again revealed that Tm1 and Tm2 show uniform and heterogeneous connectivity patterns to their presynaptic cell types, but are connected uniformly to more cell types than Tm9 [10]. It is tempting to speculate that this difference accounts for the differences in their physiological, functional variability.

Different connectivity patterns leading to the same functional properties (e.g., Tm1, Tm2), might not be functionally relevant. Additionally, the recently discovered retinal movements could help to distribute the visual stimuli across the columnar sparsity [95]. Alternatively, the heterogeneous presynaptic partners could provide relevant input, but different presynaptic circuit motifs may result in the same downstream function, i.e. the circuitry would be degenerate. Examples of degeneracy, where different components lead to the same, or very similar function, exist throughout the nervous system [96, 97, 98]. Especially many different sets of ion channels have been shown to produce the same functional outcome in a neuron, such as its firing pattern [97]. It is tempting to draw an analogy between the heterogeneous expression of ion channels in a given neuron, and the heterogeneous connectivity that emerges from connectomes. Such a degenerate design would allow for increased developmental robustness, since different connectivity can lead to the same functional outcome. At the same time, different circuit motifs might be repurposed in other contexts, simultaneously increasing the coding flexibility of the system.

Although not analyzed at the same depth, other cell types also manifest heterogeneity in the number of their synaptic connections: Mi4 vs Mi9, or Pm2a vs Pm2b, anatomically similar cell type pairs, show substantial heterogeneity in the number of connections with their primary inputs or outputs, with connection numbers varying within an order of magnitude [32]. Overall, a picture emerges in which cell types in the visual system display different degrees of heterogeneity. However, it is unclear if visual cell types will fall into “more or less heterogeneous” categories, or whether there is a continuum of connectivity patterns, and how the different conceptual possibilities for heterogeneous connectivity (Figure 4) synergize.

### 2.2 Origins of Synaptic Heterogeneity

Where does the heterogeneity in the above‐described circuit motives originate from? The visual system has been one of the systems in which the role of specific gene products defining synaptic specificity between two neuronal cell types has been extensively studied (recent reviews on visual system connectivity in [1, 99]). In addition, non‐genetic factors that contribute to wiring variability across the visual system certainly exist. For instance, changes in neuronal shape and wiring in the visual system have been attributed to visual experience [100, 101]. Furthermore, neuronal morphology—and hence, also synaptic connectivity—can differ across the course of the day and appears to be under circadian control [100, 102]. Most notably, synaptic connectivity differs substantially depending on the ambient temperature at which the fly nervous system develops. An inverse relationship between synapse numbers and developmental temperature was shown for fly photoreceptors and their postsynaptic partners [76]. A similar relationship was revealed for neurons along the olfactory pathway [103], arguing that this might be a general phenomenon, at least for sensory systems. A better understanding of such nongenetic contributions will help to explain why connectivity is different between individuals.

However, how can wiring variability between two identical neurons of the same cell types within the same fly be explained, since they have (largely) experienced the same temperature and the same lighting conditions? Synapse formation is not entirely hard‐wired, and developing neurons are quite promiscuous when choosing synaptic partners (read [104] for a more extensive review). In brief, stochastic cellular processes during development can bias synaptogenic decisions among several potential synaptic partners, by regulating membrane dynamics, synaptic competency, and selective adhesion between putative partners [104]. At the cellular level, spatiotemporal separation (for instance, via the formation of layers or columns) can reduce the chance of two partner neurons ever meeting. At the subcellular layer, dynamic filopodia at the tip of a growing axon, the growth cone, then explore the environment in search of potential synaptic partners. A differential encounter with the postsynaptic membrane of one cell, but not another cell, can lead to the stabilization of these contacts. The formation of specific synapses will be initiated if enough synaptic material can be recruited to this site (competency). This process can be further biased via the selective adhesion between two potential partners [104]. A detailed mechanism for spatiotemporal restriction, followed by probabilistic accumulation of building material has recently been shown to help stabilize entire branches of a class of visual projection neurons, the LC14 neurons [105]. This mechanism can thus shift the formation of synapses to a different region in the visual system, with different potential postsynaptic partners being able to form synapses with an arriving branch. A competition for limited resources leads to a probabilistic choice in branch stabilization and synapse formation, such that the resulting pre‐ and a post‐synaptic partner pairs will differ between cells of the same fly eye. Environmental factors such as developmental temperature will affect these processes, since filopodial dynamics or the availability and distribution of building material at specific membrane sites depend on temperature. Overall, both environmental and cell‐intrinsic factors and their interplay will result in variable wiring patterns in and across visual systems. Thus, the outcome of such genetically encoded, yet non‐deterministic and stochastic molecular events can be a variable pattern of synaptic connectivity across the different units of the fly eye, as observed in the connectome.

## 3 Conclusions

Where do we go from here? Building on previous work that had already characterized stochasticity in visual system properties [106], recent connectomes have reveaeled widespread heterogeneity in the connectivity of cells belonging to one type. Yet, it is still early days to fully comprehend the wiring heterogeneity in the visual system, let alone its functional consequences. Future analysis will reveal whether the degree and specific type of heterogeneity might vary between cell types: some might draw from a wider distribution of synaptic partners (with whom to connect?), whereas others might be more heterogeneous in the spatial arrangement of synapses (where to connect?). It is also unclear whether there is coordination between cell‐type specific heterogeneity, as described above, and regional specializations. One could, for example, imagine that systems which afford heterogeneity on the front end, such as the color‐vision system with its stochastic ommatidial pattern, impose more constraints on the underlying downstream circuitry than a system with a homogeneous front end. To answer these questions, we will need to analyze more cell types at a level of detail that has just become possible with the recently released connectomes. Furthermore, the comparison between existing and future connectomes, electron microscopy or light microscopy based, will allow us to determine how heterogeneity itself changes across individuals, or depends on external factors.

Ultimately, one of course wants to understand how heterogeneity relates to function. In general, visual processing is adapted to the stimulus statistics across the visual field, which are non‐uniform. Whether the heterogeneities described here help accommodate these statistics, and are ethologically relevant, is not understood. If different connectivity patterns lead to functional diversity, then not every unit of the visual system will process the same information. This might challenge translational invariance at first glance. However, especially global visual input patterns will remain behaviorally meaningful if not every retinotopic unit contributes. Alternatively, many examples in biology point to a degenerate code for neuronal function. In that sense, different connectivity patterns might result in the same function of a neuron, thereby preserving translational invariance. Irrespective of the functional consequences that will emerge in the future, heterogeneity in synaptic connectivity argues for “imprecision in vision”.

## Author Contributions

**M. F. W**. and **M. S**. both conceptualized, wrote and edited the manuscripts, and acquired funding.

## Conflicts of Interest

All authors have no conflicts of interest.

## Data Availability

Code related to the production of figures in this article is available at github.com/Silieslab/Bioessays_2026_WernetSilies.
